# Relationship Between Hand Kinematics, Hand Hydrodynamic Pressure Distribution and Hand Propulsive Force in Sprint Front Crawl Swimming

**DOI:** 10.3389/fspor.2022.786459

**Published:** 2022-02-15

**Authors:** Daiki Koga, Takaaki Tsunokawa, Yasuo Sengoku, Kenta Homoto, Yusaku Nakazono, Hideki Takagi

**Affiliations:** ^1^Graduate School of Comprehensive Human Sciences, University of Tsukuba, Tsukuba, Japan; ^2^Faculty of Health and Sport Sciences, University of Tsukuba, Tsukuba, Japan

**Keywords:** hand propulsive force, swimming velocity, hydrodynamic pressure, dorsum pressure, stroke frequency

## Abstract

**Purpose:**

This study investigated the relationship between hand kinematics, hand hydrodynamic pressure distribution and hand propulsive force when swimming the front crawl with maximum effort.

**Methods:**

Twenty-four male swimmers participated in the study, and the competition levels ranged from regional to national finals. The trials consisted of three 20 m front crawl swims with apnea and maximal effort, one of which was selected for analysis. Six small pressure sensors were attached to each hand to measure the hydrodynamic pressure distribution in the hands, 15 motion capture cameras were placed in the water to obtain the actual coordinates of the hands.

**Results:**

Mean swimming velocity was positively correlated with hand speed (*r* = 0.881), propulsive force (*r* = 0.751) and pressure force (*r* = 0.687). Pressure on the dorsum of the hand showed very high and high negative correlations with hand speed (*r* = −0.720), propulsive force (*r* = −0.656) and mean swimming velocity (*r* = −0.676). On the contrary, palm pressure did not correlate with hand speed and mean swimming velocity. Still, it showed positive correlations with propulsive force (*r* = 0.512), pressure force (*r* = 0.736) and angle of attack (*r* = 0.471). Comparing the absolute values of the mean pressure on the palm and the dorsum of the hand, the mean pressure on the dorsum was significantly higher and had a larger effect size (*d* = 3.71).

**Conclusion:**

It is suggested that higher hand speed resulted in a more significant decrease in dorsum pressure (absolute value greater than palm pressure), increasing the hand propulsive force and improving mean swimming velocity.

## Introduction

Mainly two factors determine the swimming velocity: propulsion and drag force. When the swimming velocity is constant, the mean propulsion and the mean drag are the same (van der Vaart et al., 1987). Increasing the swimming velocity requires increasing the propulsion or decreasing the drag. However, due to the complexity of unsteady flow mechanics in human swimming, it is currently impossible to measure propulsion and drag directly. Thus, researchers have established indirect methods to estimate these forces, such as the MAD-system (Hollander et al., 1986), velocity perturbation method (Kolmogorov and Duplishcheva, 1992), assisted towing method (Formosa et al., 2012), MRT (measured values of residual thrust) method (Narita et al., 2017). These methods enable researchers to estimate drag (and consequently propulsion, assuming the swimmer maintains a constant velocity ignoring force and velocity fluctuations within a stroke cycle) acting on the whole body but do not provide information on the sources of the total forces.

On the other hand, pressure sensors have been used to estimate the propulsion exerted by the hand in recent years (Kudo et al., 2012; Tsunokawa et al., 2019a; Koga et al., 2020, 2021). The measurement method using pressure sensors has a limitation: it can only measure the fluid force exerted by a part of the swimmer's body. On the other hand, it can directly identify the magnitude of the force and the direction of force acting in real-time. Because of the above, it is more realistic to use propulsion rather than drag as a cue to obtain empirical data for improving swimming velocity.

It has been suggested that the arms exert more propulsion than the legs in front crawl swimming (Cohen et al., 2017) and that the hands contribute the most propulsion among the upper arm, forearm and hand (Toussaint et al., 2002; Samson et al., 2017; Takagi et al., 2021). Hence, the magnitude of propulsion in hand is related to swimming velocity (Tsunokawa et al., 2019b). Kudo et al. (2016) compared the hand propulsive force in the Insweep and Upsweep phases during 25 m front crawl swimming with a maximum effort by advanced and intermediate level swimmers. The results showed that advanced swimmers exhibited more significant hand propulsive force, and a higher competitive level was associated with more substantial hand propulsive force.

The forces acting on the body surface underwater include pressure and friction. Since pressure is the major contributor to hand propulsive force (Samson et al., 2017), the hand propulsive force is calculated as the force in the propulsive direction by measuring the pressure on the hand surface (Tsunokawa et al., 2018a,b). The pressure force of the hand is calculated by multiplying the (so called) hydrodynamic pressure difference between hydrodynamic pressure on the palm side and dorsum side of the hand by the hand's area. The hydrodynamic pressure difference is related to the magnitude of the hand pressure force because the hydrodynamic pressure acts from the higher pressure to the lower pressure. In front crawl swimming, hydrodynamic pressure on the palm side shows a positive value, while the hydrodynamic pressure on the dorsum side shows a negative value (Takagi et al., 2014). In a study investigating the change in hand pressure force with increasing stroke frequency in front crawl swimming, the hand pressure force increased with increasing stroke frequency. The increase in hand pressure force was due to the more significant contribution from the increase in absolute hydrodynamic pressure on the dorsum side than on the palm side (Koga et al., 2021). However, this study reported hydrodynamic pressure distributions within individuals and cycles. Still, the relationship between the magnitude of the propulsive force and the value of hydrodynamic pressure distribution between individuals was not clarified.

In addition, it has been reported that the magnitude of hand propulsive force varied with some kinematic variables. A study that subjectively and gradually increased swimming velocity reported an increase in hand propulsive force, as well as an increase in stroke frequency and hand speed (Tsunokawa et al., 2019a). In a study in which stroke frequency was increased to over self-selected stroke frequency, both hand propulsive force and angle of attack decreased (Koga et al., 2020). This decrease of attack angle has been suggested to be related to the value of hydrodynamic pressure on the palm side. Thus, it is inferred that some kinematics of the hand affect the magnitude of hand propulsive force.

However, previous studies have not clarified the relationship between the kinematic variables of the hand, the hand's pressure distribution, and the fluid force exerted by the hand when swimming the front crawl. Therefore, this study aimed to determine the interrelationships between the hand kinematic variables, hydrodynamic pressure, and fluid forces exerted by trained swimmers when swimming the front crawl with maximum effort. The results obtained are expected to provide coaches and swimmers with new insights into the mechanisms of what they should keep in mind to swim faster.

## Materials and Methods

### Participant

Twenty-four male swimmers participated in this study, and their competition level ranged from the regional to the national final. The personal characteristics of the swimmers are shown in Table 1. The swimmers were informed purpose and content of this study and the risks involved, and their written consent to participate was obtained. The Ethics Committee approved the study of the University of Tsukuba.

**Table 1:** Participants' physical characteristics, speciality and performance level.

**Swimmer**	**Age (years)**	**Height (m)**	**Weight (kg)**	**Speciality**	**Best Record of 50 m front crawl (s")**	**FINA point**
A	19	176.0	72.0	Front crawl	22.61	791.0
B	26	174.0	72.0	Front crawl	22.79	772.4
C	26	184.0	81.0	Front crawl	22.96	755.3
D	25	181.0	76.0	Front crawl	22.98	753.4
E	22	177.0	80.0	Front crawl	23.22	730.3
F	22	187.0	80.0	Front crawl	23.27	725.6
G	24	186.0	78.0	Front crawl	23.50	704.5
H	21	169.0	69.0	Front crawl	23.52	702.7
I	21	175.0	70.0	Back stroke	23.86	673.1
J	22	174.6	79.0	Front crawl	23.93	667.2
K	20	181.0	71.5	Front crawl	24.06	656.4
L	19	175.5	70.0	Breast stroke	24.20	645.1
M	21	183.0	77.0	Front crawl	24.26	640.3
N	21	175.0	75.0	Individual medley	24.31	636.4
O	22	169.0	63.0	Back stroke	24.87	594.3
P	23	176.0	72.5	Breast stroke	24.89	592.9
Q	21	179.0	70.0	Front crawl	24.91	591.5
R	20	168.0	62.0	Butterfly	24.99	585.8
S	20	172.5	68.0	Back stroke	25.00	585.1
T	20	175.0	69.0	Front crawl	25.18	572.7
U	20	175.0	74.0	Individual medley	25.28	565.9
V	19	176.0	78.0	Back stroke	25.42	556.6
W	20	171.0	68.0	Breast stroke	25.55	548.1
X	20	164.0	66.0	Front crawl	26.24	506.0
Mean	21.4	176.0	72.5		24.24	648.0
SD	2.0	5.6	5.2		0.97	78.2

### Experimental Setup

The experiment was conducted in the indoor 50 m pool. After a self-selected warm-up, the swimmers were asked to perform three 20 m front crawl swimming trials with no breathing and maximum effort. The trial area was between 5 and 25 m from the wall, and the swimmers started in a floating position to avoid the effect of the wall kicking on their swimming velocity. One stroke cycle in front crawl swimming was defined as the duration of entry of one hand into the water to the entry of the same hand again. Due to the limitations of the measurement area, the motion capture system could not capture all markers during a complete stroke, depending on when the swimmer entered the measurement area. Therefore, the trial with an entire one-stroke cycle within the measurement area was considered the appropriate trial for analysis among the three trials.

### Data Acquisition

**Figure 1:**
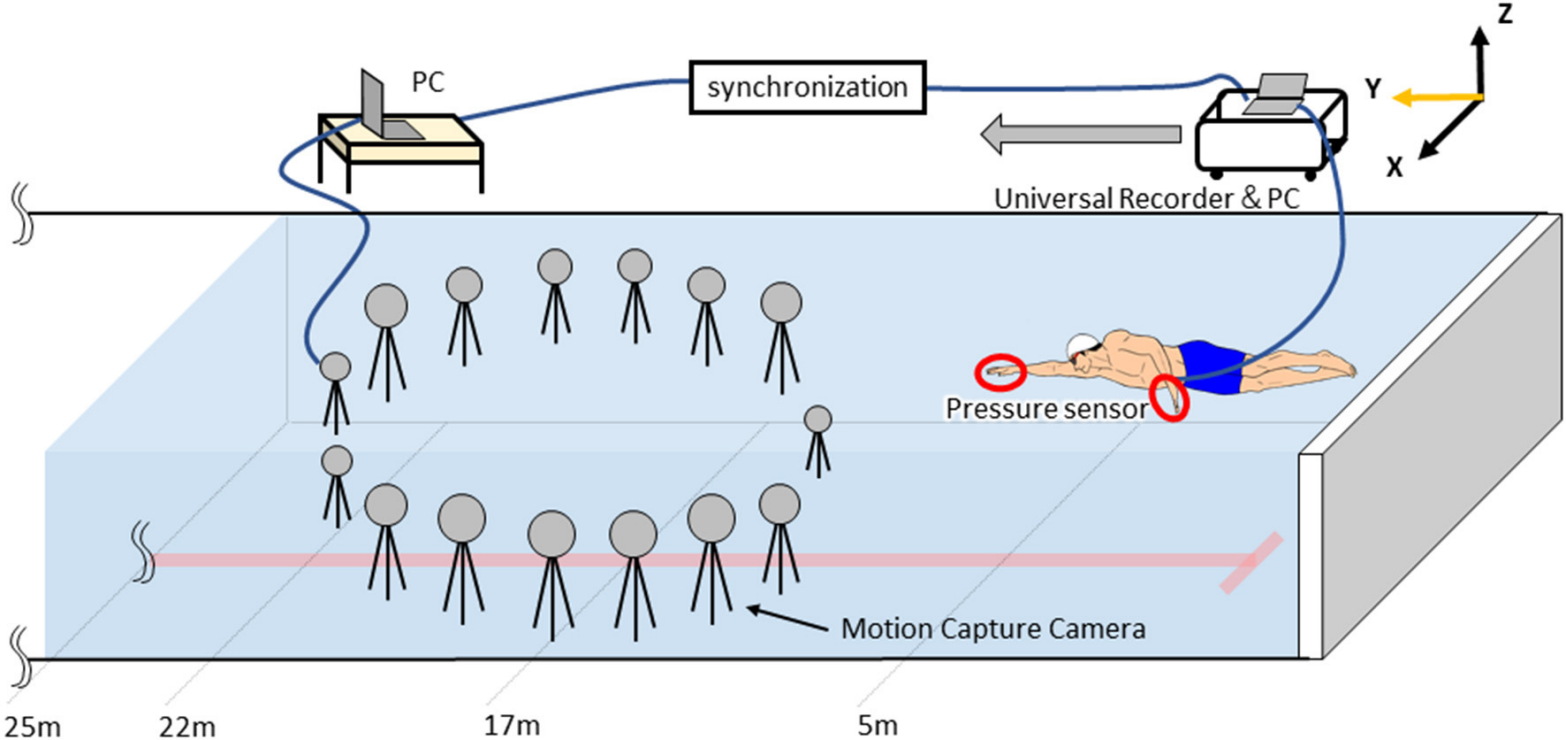
Experimental setting. Placement of motion capture cameras and measurement area.

**Figure 2:**
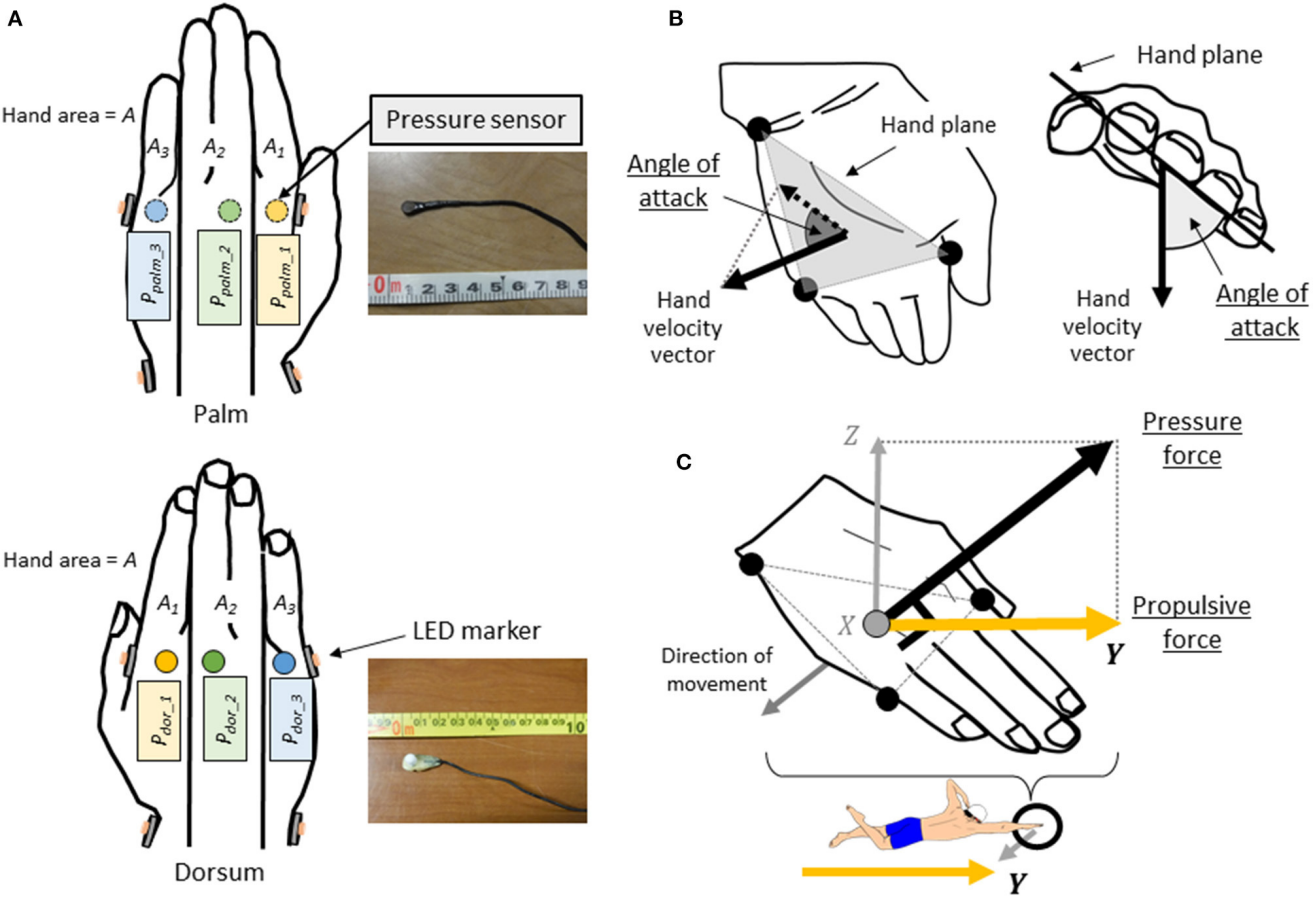
**(A)** The location of the pressure sensors and the LED markers attached to the hands. **(B)** Definition of angle of attack. **(C)** Definition of hand plane, hand pressure force acting perpendicular to the hand plane, and hand propulsive force.

Three-dimensional motion analysis was conducted using a three-dimensional real-time motion measurement system, VENUS 3D (Nobby Tech. Ltd., Japan), to obtain absolute coordinates of markers. The measurement area was 5 m between 17 and 22 m from the pool wall, and 15 cameras were placed underwater surrounding the measurement area (Figure 1). The water depth of the measurement area was 2 m. We used the dynamic calibration system provided with the VENUS 3D to acquire more than 2,000 samples by swinging the wand to calibrate the measurement area. The standard error of the underwater motion capture in calibration was <0.3 mm. LED markers were attached to 10 points on the left and right great trochanter, the left and right second and fifth metacarpophalangeal joints, the left and right radial styloid process, and the left and right ulnar styloid process (Figure 2A). The trials were recorded with 100 Hz. This study used a fixed right-hand coordinate system with the swimmer's propulsive direction as the Y-axis, the lateral directions as the X-axis, and the vertical direction as the Z-axis.

Waterproofed small pressure sensors (Round, diameter: 6 mm, thickness: 0.6 mm, PS-05KC, Kyowa Electronic Instruments Co. Ltd., Japan, Figure 2A) were attached to the swimmer's hand to measure the pressure distribution on the hands during the trial, following the method described by Tsunokawa et al. (2018b). At 12 locations, the sensors were attached to the palm and dorsum sides of the second, third and fifth metacarpophalangeal joints. The hand plane was divided into three segments (A_1_-_3_) by the second and fourth interphalangeal spaces (Area: A_1_ = 54.8 ± 11.0 cm^2^, A_2_ = 73.4 ± 8.3 cm^2^, A_3_ = 39.5 ± 6.8 cm^2^, Figure 2A). Pressure was assumed to act uniformly in a segment, and the value of each pressure sensor was defined the representative pressure value acted to the each segment. The signals output from the pressure sensors were recorded on a laptop with 100 Hz by using a universal recorder (EDX-100A, Kyowa Electronic Instruments Co. Ltd., Japan). All signals from the motion capture system and the pressure sensors were synchronized and stored on a laptop. Since the pressure sensors were wired, a cart carrying the equipment was moved with the swimmer (Figure 1). Because the motion capture cameras were placed only underwater, only the stroke motion underwater was analyzed.

### Data Analysis

#### Kinematic Parameters

The average swimming velocity per stroke cycle was calculated by time-differentiating the displacement that the midpoint of the left and right great trochanter moved in the Y-axis direction, calculated using motion capture analysis software (VESUS 3D 4.3, Nobby Tech. Ltd., Japan). The stroke frequency was calculated from the reciprocal of the time taken per cycle, and the stroke length was calculated by dividing swimming velocity by stroke frequency. The hand speed was calculated by time-differentiating the 3D displacement traveled at the midpoint of each coordinate of the hand (second and fifth metacarpophalangeal joints, ulnar styloid process). The distance traveled by the hand in the water was calculated by multiplying the speed of the hand by the time taken for one stroke in the water. The angle of attack was calculated as the angle of projection of the hand velocity vector onto the plane of the hand composed of two vectors pointing from the ulnar styloid process to the fifth and second metacarpophalangeal joints (Figure 2B). The hand speed, angle of attack, distance traveled by the hand in water and stroke time in water were averaged only during the period of movement through the water in the measurement space.

#### Hydrodynamic Pressure

**Figure 3:**
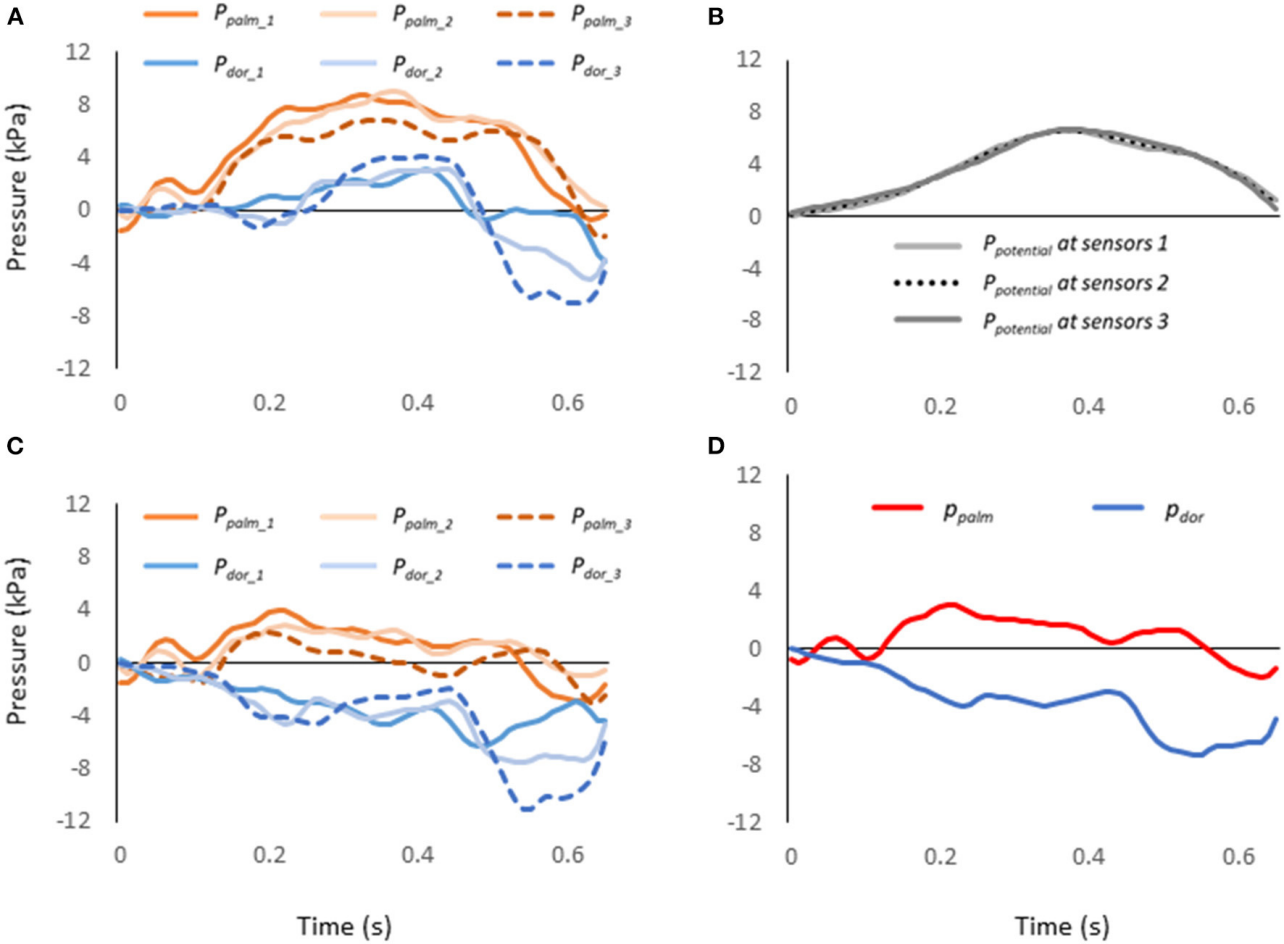
**(A)** Pressure value measured by each pressure sensor. **(B)** *P*_*potential*_ at each pressure sensor. **(C)** *P*_*effect*_ at each pressure sensor calculated by subtracting *P*_*potential*_ from the value of each pressure sensor. **(D)** The representative values of the pressure calculated considering the differences between the three segments of the hand.

The pressure value measured at sensors (*P*_*measured*_) combined hydrodynamic pressure of *P*_*effect*_ and *P*_*potential*_ (Equation 1, Figure 3A). The effective pressure (*P*_*effect*_) is the pressure acting perpendicular to the surface of the sensor, reflecting the change in energy in the fluid due to the swimming motion. The *P*_*potential*_ is the pressure due to the change in the potential, i.e. water depth (*P*_*potential*_, Equation 2).


(1) $$\begin{array}{l} P_{measured}\;=\;P_{effect}+P_{potential} \end{array}$$

(2) $$\begin{array}{l} P_{potential}=\rho gz \end{array}$$

where ρ indicates the water density (997 kg/m^3^), relative flow velocity (*v*), *g* indicates the acceleration of gravity (9.80665 m/s^2^), and *z* indicates the depth of the pressure sensor. *z* is set to zero at the water surface, and becomes positive as it gets deeper (Figure 3B). The position of each pressure sensor attached to the hand was calculated from the coordinates of the second and fifth metacarpophalangeal joints and the midpoint of both joints, assuming that the six sensors are located at approximately the same depth of water.

For the pressure distribution measurement, atmospheric pressure was set to zero. The pressure data at each hand's segment was smoothed using a low-pass Butterworth digital filter at a cut-off frequency of 15 Hz by reference to the previous study (Tsunokawa et al., 2018b). Since the magnitude of pressure force is the pressure difference between the palm and the dorsum of the hand multiplied by the hand area, it is important to show the *P*_*effect*_ for the palm and the dorsum of the hand respectively. When determining the *P*_*effect*_ on the palm side (*p*_*palm*_) and dorsum side (*p*_*dor*_), instead of averaging the *P*_*measured*_, the area of each of the three segments (Figure 2A) and the pressure due to the change in water depth (*P*_*potential*_, Figure 3B) were considered and the *p*_*palm*_and *p*_*dor*_ were calculated according to the Equations 3 and 4 (Figures 3C,D).


(3) $$p_{palm}=\frac{\sum_{i=1}^{3}(p_{palm\_i}\;-\;P_{potential\_i})\;\times \;A_{i}}{A}$$

(4) $$p_{dor}\;=\frac{\sum_{i=1}^{3}(p_{dor_{i}}-\;P_{potential\_i})\;\times \;A_{i}}{A}$$

where *A*_*i*_ indicates the hand's area of *i*-th segment (*i* = 1–3), *p*_*palm*_*i*_ and *p*_*dor*_*i*_ indicate the measured pressure on the *i*-th segment of the palm and dorsum respectively, *P*_*potential*_*i*_ indicate the pressure due to water depth on the *i*-th segment. *A* indicates the entire hand's area. Mean *p*_*palm*_ and *p*_*dor*_ were calculated only when the hand was in the underwater phase.

#### Hand Pressure Force and Propulsive Force

**Figure 4:**
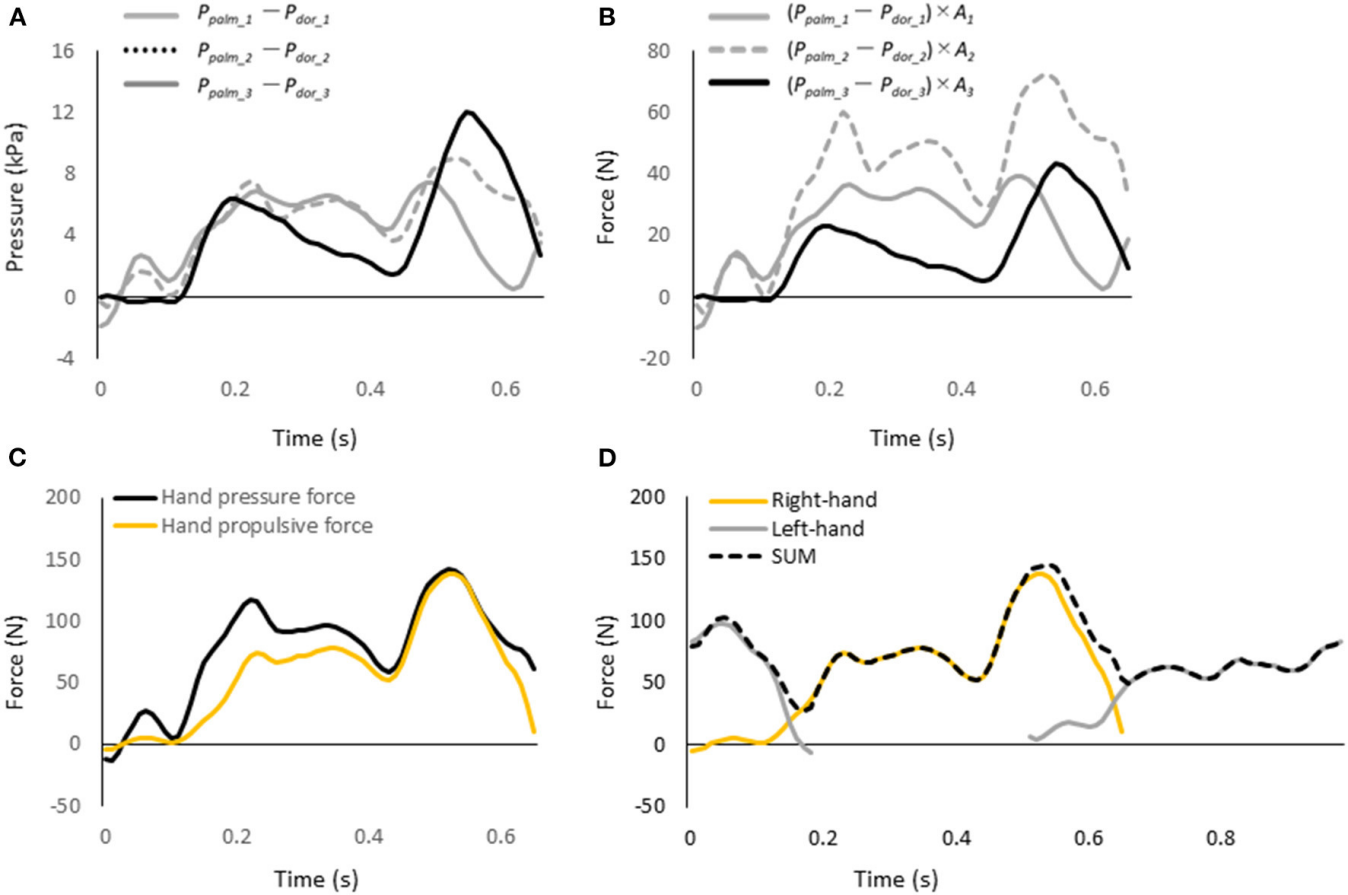
**(A)** Pressure difference in each hand segment. **(B)** Pressure force in each hand segment. **(C)** Pressure force and propulsive force exerted by the entire hand. **(D)** Hand propulsive force of the sum of the left and right values at each time point (dash line).

The hand pressure force was calculated by multiplying the difference between the pressures measured on the palm and dorsum side of the hand in each segment by the area of the segment and summing the forces in the three segments, as shown in Equation 5 (Figures 4A–C)


(5) $$\begin{array}{l} \text{Hand pressure force}=\;\sum_{i=1}^{3}\left(p_{palm\text{\_}i}-\;p_{dor\text{\_}i}\right)\times A_{i} \end{array}$$

where *A*_*i*_ indicates the hand's area of *i*-th segment (*i* = 1–3), *p*_*palm*_*i*_ and *p*_*dor*_*i*_ indicate the measured pressure on the *i*-th segment of the palm and dorsum, respectively. In the calculation of the difference between pressure on the palm and the dorsum of the hand, it is not necessary to consider the depth and the hand area, because the sensors depth and area of the hand where the pressure acts are approximately the same on the palm and the dorsum of the hand. Therefore, the pressure difference between the palm and dorsum of the hand is calculated by directly calculating the difference using the values measured by pressure sensors on the palm and dorsum, and the difference between pressure on the palm and the dorsum of the hand was calculated.

This hand pressure force refers to the hydrodynamic force acting perpendicular to the plane of the hand. Therefore, the pressure force's vector of the hand was assumed to be the same as the normal vector perpendicular to the plane of the hand (Figure 2C). The hand propulsive force is defined as the hand pressure force acting in the direction of the Y-axis, which is the propulsive direction of the swimmer. Therefore, the unit vector of each directional component of the normal vector to the hand plane was obtained, and the hand propulsive force was calculated by multiplying the hand pressure force by the unit vector in the Y-axis direction, as shown in Figure 2C.

Since the hand pressure force and propulsive force were measured with the left and right hands, the sum of the left- and right-hand values at each time point were calculated (Figure 4D). Then the average value of the hand's pressure force and propulsive force for one stroke cycle were calculated. In addition, the propulsion ratio, which indicates how much of the hand pressure force was used to propel the hand, was calculated. The propulsion ratio was calculated by dividing the propulsive force of the hand by the pressure force of the hand (Tsunokawa et al., 2018a).

### Statistical Analysis

Data for all variables were analyzed using time averages as representative values. Data were analyzed using SPSS Statistics 25.0. The normality of the samples was verified using the Shapiro-Wilk test, and the results showed that all data were normally distributed. The Pearson correlation coefficient was calculated to test the relationship between each variable. The coefficient of correlation <0.30 indicated a low correlation, between 0.31 and 0.49 indicated a moderate correlation, between 0.50 and 0.69 indicated high correlation, between 0.70 and 0.89 indicated a very high correlation, and higher than 0.90 indicate extremely high correlation (Hopkins et al., 2009). In addition, a unpaired *t*-test was used to compare the mean absolute value of the *p*_*palm*_ and *p*_*dor*_. Cohen's d was used to calculate the effect size. Cohen's d value <0.60 indicated a small effect size, between 0.61 and 1.20 indicated a moderate effect size, between 1.21 and 2.00 indicated a large effect size, between 2.01 and 4.00 indicated a very large effect size, and higher than 4.01 indicated a extremely large effect size (Hopkins et al., 2009; Barbosa et al., 2021). The statistical significance level was set at α = 0.05.

## Results

**Figure 5:**
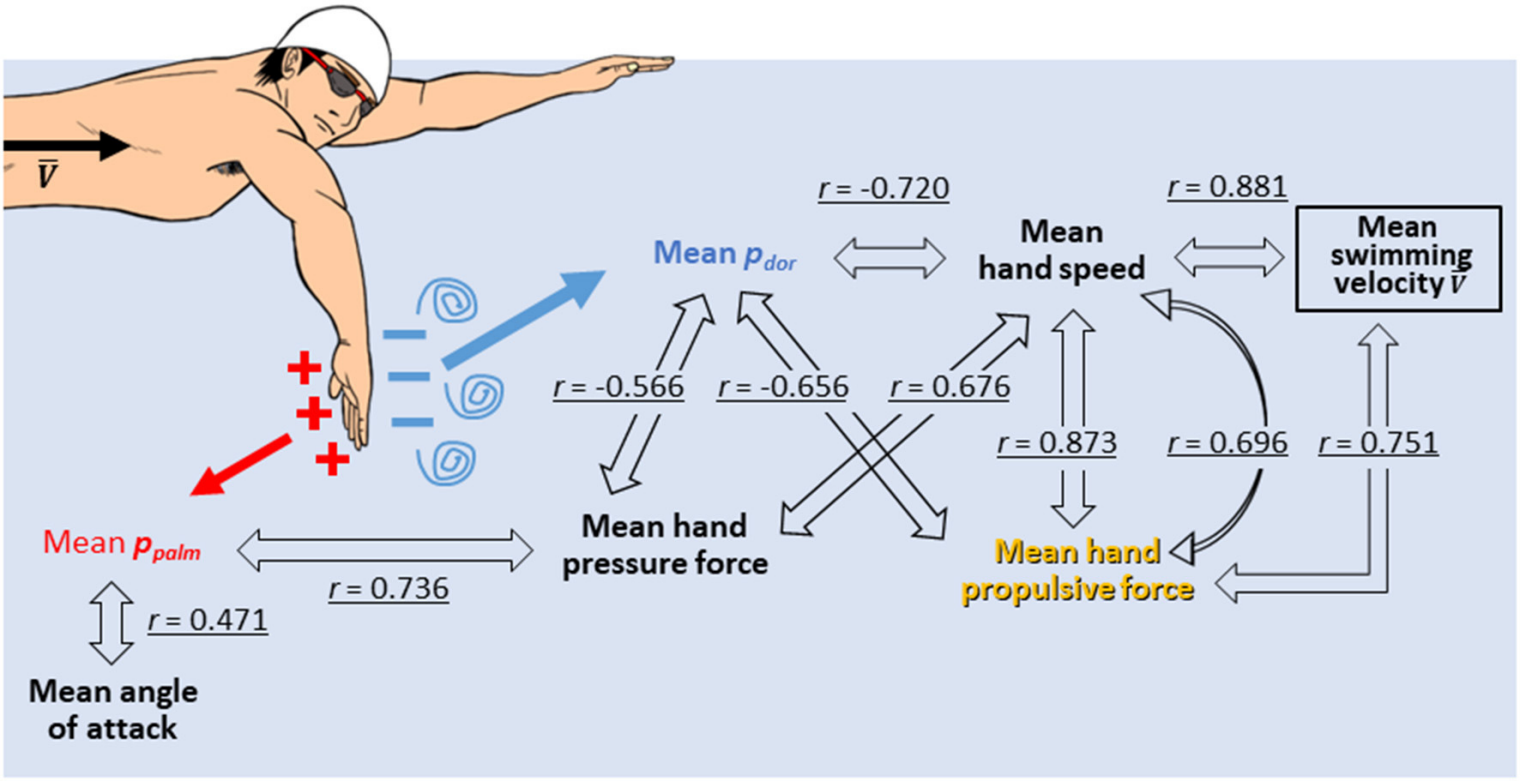
Schematic representation of the mutual correlation coefficients for each variable.

Correlation coefficients between the variables are shown in Table 2, and variables with a statistical significance level of <5% were highlighted in gray. In addition, Figure 5 shows a schematic representation of the mutual correlation coefficients for each variable.

**Table 2:**
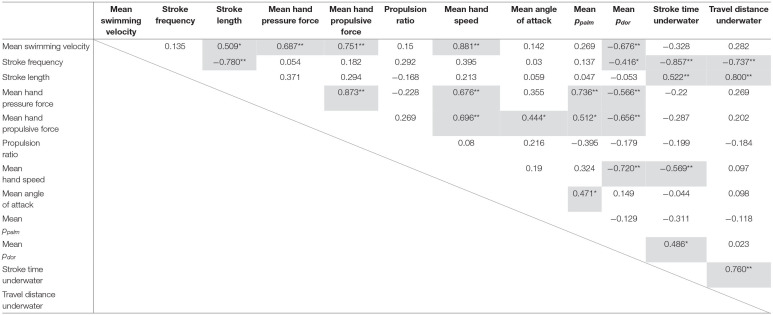
Correlation coefficient of each variable.

*p < 0.05, **.p < 0.01. Gray shades indicate that the correlation is significant.

**Figure 6:**
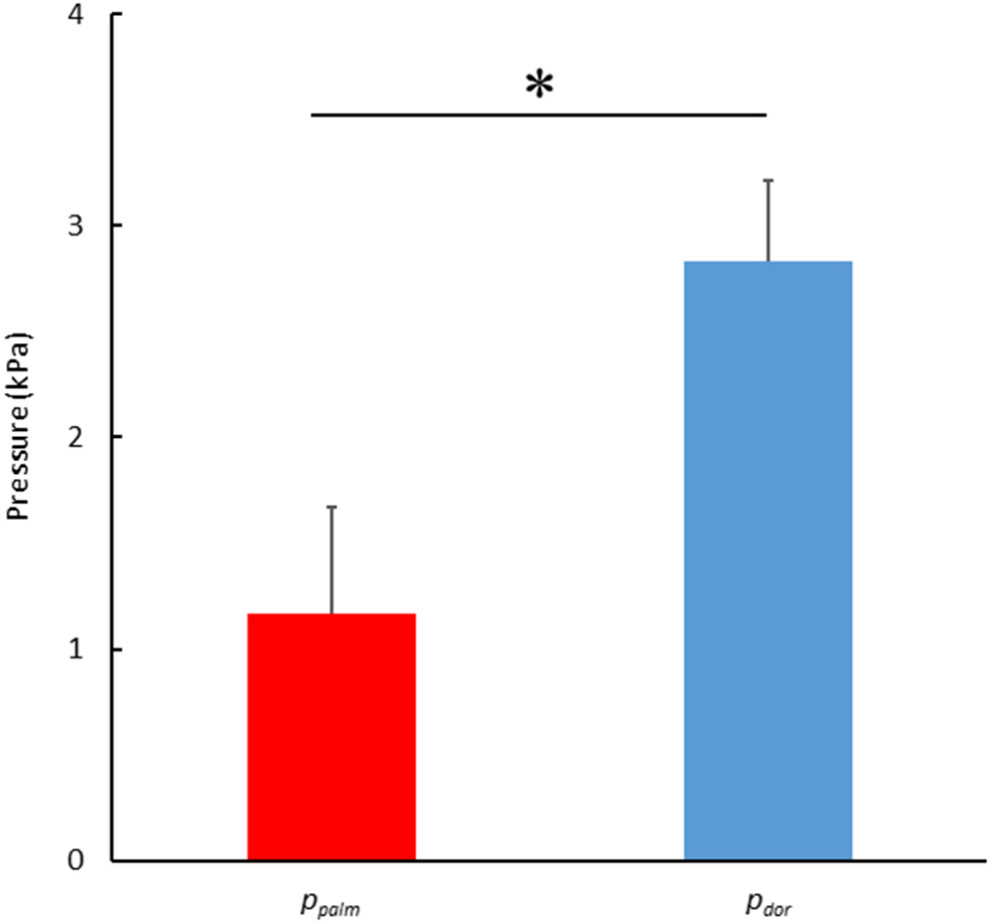
Comparison of the mean pressure in the absolute value between the *p*_*palm*_ and the *p*_*dor*_. (*p < 0.05).

Figure 6 shows the results of comparing the mean absolute values of *p*_*palm*_ and *p*_*dor*_. The absolute values of the *p*_*dor*_ were significantly higher than the *p*_*palm*_ (*p*_*palm*_: 1.17 ± 0.50 kPa, *p*_*dor*_: 2.83 ± 0.39 kPa, *p* < 0.001, effect size 3.71).

**Figure 7:**
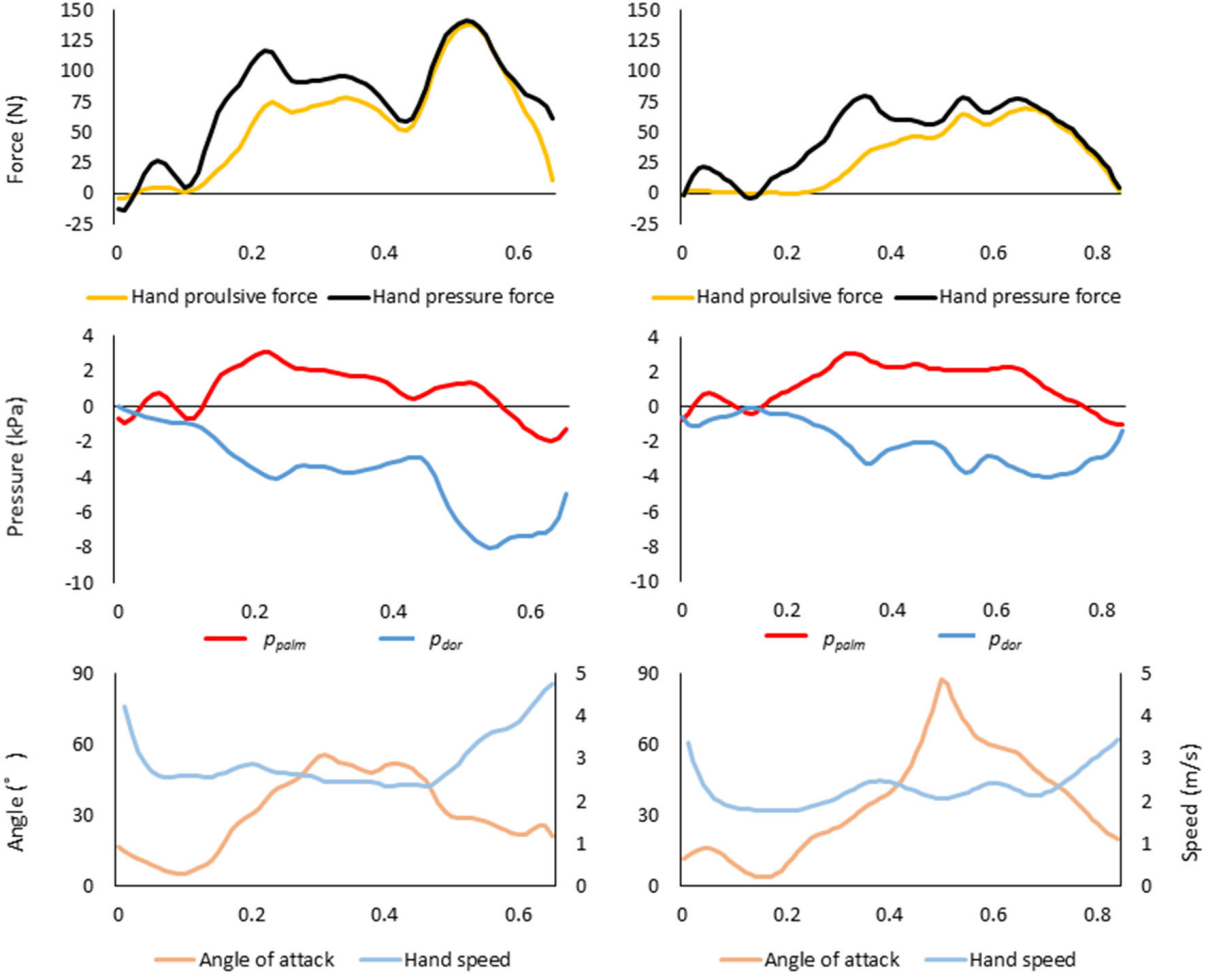
Variation over time of each variable (top, fluid force; middle, effective pressure; bottom, angle of attack and hand speed) in the right hand of the fastest swimmer A (left column) and the slowest swimmer X (right column).

Figure 7 shows the changes in the right-hand overtime variables for the fastest swimmer A and the slowest swimmer X as a typical example. Compared to swimmer X, swimmer A had greater hand propulsive force and hand pressure force, the higher absolute value of *p*_*dor*_, and higher hand speed.

## Discussion

This study aimed to identify the factors responsible for high hand propulsive force in swimmers who can reach high swimming velocity and assess the influence of hand hydrodynamic pressure and hand kinematics. Swimming velocity was significantly positively correlated with hand speed, propulsive force and pressure force, with correlation coefficients of *r* = 0.881, 0.751 and 0.687, respectively. Understandably, the swimming velocity showed a high or very high correlation with the above variables. In other words, the faster the hand moves in the water, the greater the hydrodynamic force exerted by the hand is. Therefore, the greater the propulsive force acting in the propulsive direction greater the swimming velocity, a very straightforward mechanism. However, although this mechanism is based on the interpretation of the quasi-steady theory, recent studies (Toussaint et al., 2002; Takagi et al., 2014) have shown that the quasi-steady assumption does not necessarily hold because the flow field around the hand is unsteady. Therefore, based on the results of this study, we will discuss the mechanism of improving propulsion and swimming velocity.

The pressure force was calculated by multiplying the difference between the pressures measured on the palm and dorsum side of the hand by the hand's area. Thus, the greater the pressure difference, the greater the pressure force exerted. Therefore, the *p*_*palm*_ and *p*_*dor*_ were considered separately. Firstly, the *p*_*dor*_ showed a very high and high negative correlation with the hand speed (*r* = −0.720), the propulsive force (*r* = −0.656) and the swimming velocity (*r* = −0.676) (Table 2). In other words, it can be interpreted that as the hand speed increased, the *p*_*dor*_ decreased, and the propulsive force increased, which led to an increase in swimming velocity. On the other hand, *p*_*palm*_ was not correlated with hand speed but showed a positive correlation with propulsive force (*r* = 0.512) and angle of attack (*r* = 0.471) (Table 2). Furthermore, a comparison of the absolute values of *p*_*palm*_ and *p*_*dor*_ shows that the absolute value on the *P*_*dor*_ was more than twice as large as it on the *p*_*palm*_, as shown in Figure 6.

Integrating the above results assumes that the swimmer with high hand speed generates a strong vortex on the dorsum side of the hand, which reduces the *p*_*dor*_ (Takagi et al., 2014), resulting in a negative pressure, and the absolute value of which is considerably greater than the *p*_*palm*_. This significant decrease in *p*_*dor*_ increases the hydrodynamic pressure difference between the palm and dorsum of the hand. It is main contributer to the increase in hydrodynamic force. This phenomenon was also confirmed in the work of Takagi et al. (2013, 2014), who analyzed the flow around the hand using PIV (Particle Image Velocimetry) with a robotic arm. They reported that vortices were generated on the dorsum side of the hand, especially at the point where the direction of movement of the hand changed, resulting in an unsteady lift force were reported. A study that observed the behavior of water around the hand through simple demonstrations also suggest that the force generation at the hand is primarily due to the acceleration of water on the dorsum of the hand, rather than “pushing” water on the palm of the hand (Soh and Sanders, 2021). In addition, Fuchiwaki et al. (2007) investigated the vortex structure of the wake of a wing undergoing heaving motion at different motion speeds. They reported that the higher the motion speed, the higher the vorticity of the wake and the higher the propulsive force. A reduction in the *p*_*dor*_ is more critical than *p*_*palm*_, but this does not mean the *p*_*palm*_ is not involved in the increase in propulsive force. The *p*_*palm*_ is also related to pressure force and propulsive force but is less involved than the *p*_*dor*_, suggesting that the *p*_*dor*_ is more important for faster swimmers. This suggestion is shared by the results obtained in the Koga et al. (2021) experiment, which analyzed *p*_*palm*_ and *p*_*dor*_ changes in well-trained swimmers by gradually increasing the stroke frequency within individuals. This study reported that as the stroke frequency was increased, the swimming velocity also increased, but the *p*_*palm*_ did not increase significantly, whereas the *p*_*dor*_ decreased significantly and the hydrodynamic pressure difference increased. This phenomenon is consistent with the report of Tsunokawa et al. (2015) that the absolute value of the *p*_*dor*_ of the foot was higher than the *p*_*palm*_ of the foot during the breaststroke kicking without upper limb movement. Moreover, it is consistent with Kawai et al. (2020) report investigating the foot propulsive force and hydrodynamic pressure distribution during the eggbeater kicking with maximum effort in water polo players. They reported that the variation of the magnitude of the foot pressure force was more in tune with the value of *p*_*dor*_ of the foot than the *p*_*palm*_ of the foot.

However, this fact may be hard to believe for swimmers who have always thought to push the water to move forward. Therefore, we would like to suggest some hints for improving swimming velocity by comparing the raw data of swimmer A, who had the highest average swimming velocity, and swimmer X, who had the slowest average swimming velocity, among the 24 swimmers who participated in this experiment (Figure 7). In the upper part of Figure 7, the pressure force (black) and the propulsive force (yellow) during one stroke are shown. It is clear from the figure that the pressure force and propulsive force of swimmer A were larger than those of swimmer X from the middle to the end of the stroke. Next, focusing on the *p*_*palm*_ (red) and the *p*_*dor*_ (blue) in the middle row, the *p*_*dor*_ of swimmer A was much lower than that of swimmer X, although the *p*_*palm*_ were similar. This gap between the *p*_*palm*_ and *p*_*dor*_ is directly related to the pressure force and the propulsive force, so it can be inferred that the propulsive force of swimmer X is lower as a result. Finally, comparing the hand speed (light blue) and the angle of attack (orange), the hand speed of swimmer A increased significantly in the second half of the stroke, whereas the speed of swimmer X did not increase much. Swimmer A's angle of attack ranged from 20° to 50°, whereas Swimmer X's angle of attack varied considerably from 10° to 90°. Since the attack angle is correlated with the pressure on the palm, it should be kept to a certain degree. However, the CFD (Computer Fluid Dynamics) flow visualization experiment by Samson et al. (2018) showed that either too large or too small an angle of attack might not cause a decrease in pressure on the dorsum of the hand effectively. Therefore, it may be advisable to maintain an optimal angle of attack.

Next, we discuss the relationship between the swimming velocity and hand kinematics and stroke indices. As mentioned earlier, the swimming velocity was highly correlated with the hand speed. However, the hand speed did not correlate with stroke frequency (Table 2). The result above mentioned is an essential point. If a swimmer blindly increases the number of strokes to increase hand speed, the time and distance that the hand moves in the water will be shortened, and the hand may leave the water without increasing hand speed sufficiently. Stroke length was also correlated with swimming velocity (*r* = 0.509). In other words, if the swimmer swam faster, the distance traveled in one stroke would be longer, but stroke length is only a result, and a longer stroke length did not imply a faster swim speed. Instead, it should be understood that the distance traveled per stroke was longer due to moving the hand as fast and long as possible in the water. So how can swimmers increase the speed of their hands? Although we cannot draw any conclusions based on the results of this study alone, a possible inference is that swimmers need to consider the following factors to increase hand speed. For example, a combination of left and right upper limbs, rolling movements, and coordination of upper limb stroking movements with lower limb kicking movements. Because, unlike on land, there is no fulcrum in the water, so simply trying to increase hand speed may not result in the desired increase in speed.

For the relationship between the swimming velocity and hand forces, the swimming velocity was highly correlated with the hand's pressure and propulsive force. The hand pressure force was also highly correlated (*r* = 0.873) with the hand propulsive force. However, there was no relationship between the swimming velocity and propulsive ratio. In other words, swimmers with high swimming velocity do not necessarily have a higher propulsion ratio. In front crawl swimming, the force exerted by the hand acts not only in the propulsive direction but also in the vertical and lateral directions. These back and forth, vertical and horizontal fluid forces are thought to have different roles. For example, the force acting vertically upward lifts the body near or above the water's surface (Nakashima, 2007) and may reduce the area that receives drag from the water. Therefore, to swim faster, the hand pressure force should act not only in the propulsive direction but also in the vertical and lateral directions. Because the forces acting vertical and lateral direction might contribute to lifting the body, reducing resistance and promoting a rolling motion of the upper trunk would increase the speed of the backward movement of the hand.

### Limitation and Future Tasks

This study has some limitations. The pressure sensor used in the hydrodynamic pressure distribution measurement can only measure the hydrodynamic pressure acting perpendicular to the hand plane. It has been suggested that the negative hydrodynamic pressure increases in the latter half of the underwater stroke due to the effect of frictional forces caused by the generation of axial flow from the shoulder to the hand (Toussaint et al., 2002). Therefore, it is necessary to measure the friction component to measure the propulsive force accurately. However, considering that the main cause of hand propulsive force is the pressure component (Samson et al., 2017), estimating the propulsive force by measuring the hand's hydrodynamic pressure distribution seems reasonable.

In this study, the pressure was assumed to act uniformly on the each hand segments, and the value of each pressure sensor was defined as the representative pressure value acting on each segment. In reality, the value varies depending on the part of the hand surface. Hence, more sensors need to be used to subdivide the hand segments and improve the accuracy of the measurement. However, at present, the sensors are wired, and affixing many sensors to both hands may interfere with the swimming motion. Therefore, there is a need to develop a measurement method that provides both high measurement accuracy and less burden to the swimmer.

In front crawl swimming, the arms exert greater propulsive force than the legs (Cohen et al., 2017), and of the upper arms, forearms and hands, it has been suggested that the hands exert the largest propulsive force (Toussaint et al., 2002; Samson et al., 2017; Takagi et al., 2021). Therefore, the present experiment was conducted based on the assumption that the force exerted by the segments other than the hand would be negligible. However, the results of this experiment showed that the rolling and kicking movements are also essential factors in increasing hand speed. Silveira et al. (2017) also reported that the kicking motion by the lower lims increases stroke length, which in turn affects swimming velocity. The next step is to take a more macroscopic view of the swimming motion and elucidate how hand speed is increased to obtain significant hand propulsive force.

## Conclusion

Swimmers with faster swimming velocity had higher hand speed and greater hand propulsive force. Pressure on the dorsum of the hand had a significant negative correlation with swimming velocity, hand speed and hand propulsive force. In contrast, palm pressure was not significantly correlated with swimming velocity and hand speed but was significantly correlated with hand propulsive force and angle of attack. Comparing the values of palm and dorsum pressure in absolute value, dorsum pressure was more than twice as high as palm pressure, suggesting that it significantly influences the force acting on the hand. Therefore, it can be inferred that swimmers who swim faster have a greater decrease in hand dorsum pressure due to their faster hand speed, which exerts a more significant hand propulsive force.

## Data Availability Statement

The datasets supporting the conclusions of this article will be made available by the authors without undue reservation.

## Ethics Statement

The studies involving human participants were reviewed and approved by the Ethics Committee of University of Tsukuba. The patients/participants provided their written informed consent to participate in this study.

## Author Contributions

DK, TT, YS, KH, and HT contributed to the conception and design of this study. DK, KH, and YN made data acquisition. DK wrote the manuscript draft. TT, YS, KH, and HT contributed to the manuscript revisions. All authors approved the submission of this final draft.

## Funding

This work was supported by JSPS KAKENHI (Grant No. JP 20H04064).

## Conflict of Interest

The authors declare that the research was conducted in the absence of any commercial or financial relationships that could be construed as a potential conflict of interest.

## Publisher's Note

All claims expressed in this article are solely those of the authors and do not necessarily represent those of their affiliated organizations, or those of the publisher, the editors and the reviewers. Any product that may be evaluated in this article, or claim that may be made by its manufacturer, is not guaranteed or endorsed by the publisher.
